# Editorial: Pathophysiology and Pathogenic Mechanisms of Pulmonary Vascular Disease

**DOI:** 10.3389/fphys.2022.854265

**Published:** 2022-03-18

**Authors:** Jinsheng Zhu, Jiwang Chen, Jian Wang, Ankit A. Desai, Stephen M. Black, Haiyang Tang

**Affiliations:** ^1^State Key Laboratory of Respiratory Disease, Guangdong Key Laboratory of Vascular Disease, National Clinical Research Center for Respiratory Disease, Guangzhou Institute of Respiratory Health, The First Affiliated Hospital of Guangzhou Medical University, Guangzhou, China; ^2^College of Veterinary Medicine, Northwest A&F University, Xianyang, China; ^3^Department of Medicine, University of Illinois at Chicago, Chicago, IL, United States; ^4^Cardiovascular Research Center, University of Illinois at Chicago, Chicago, IL, United States; ^5^Department of Medicine, Krannert Institute of Cardiology, Indiana University, Indianapolis, IN, United States; ^6^Department of Cellular Biology & Pharmacology, Herbert Wertheim College of Medicine, Miami, FL, United States; ^7^Department of Environmental Health Sciences, Robert Stempel College of Public Health and Social Work, University Park, FL, United States; ^8^Center for Translational Science, Florida International University, Port St. Lucie, FL, United States

**Keywords:** pulmonary hypertension, pulmonary vascular disease, pulmonary arterial hypertension, pulmonary vascular remodeling, pulmonary vascular resistance

Pulmonary vascular disease (PVD) is a general term for a variety of diseases caused by dysfunction and pathological changes in pulmonary arteries, pulmonary veins, pulmonary capillaries, and systemic lung vessels, including, but not limited to, pulmonary hypertension (PH), venous thromboembolism (VTE), pulmonary arteriovenous malformation and pulmonary vasculitis (Cummings and Bhalla, 2015). Among them, pulmonary arterial hypertension (PAH) is a rare, but devastating disease characterized by a progressive rise in pulmonary vascular resistance (PVR) and pulmonary arterial pressure (PAP). Advanced stages of PAH can lead to right heart failure and death. A central objective of this research focus is to provide new insights into the pathophysiology and pathogenesis of PVD based on the latest research advances in cellular and molecular mechanisms, animal models, and clinical studies on the development and progression of the disease. The Research Topic also includes the latest research on medical imaging as well as discovery of novel molecular targets and therapeutic drugs for PAH.

Non-invasive imaging modalities are used in the diagnosis of patients with suspected or established PH due to their ease of acquisition, safety profile, and relative accuracy. These imaging modalities include, but are not limited to, transthoracic Doppler echocardiography (TTDE), chest radiography, computed tomography (CT), radionuclide ventilation-perfusion (V/Q) lung scintigraphy, and magnetic resonance imaging (MRI). In rodent models of PH, echocardiography is commonly used to characterize right ventricular structure and function, also providing clues about the pulmonary circulation (Zhu et al., 2019). Intravascular ultrasound (IVUS), a method for combining non-invasive ultrasound imaging with invasive catheter, can be performed simultaneously with a right heart catheter, which reflects intravascular conditions in real-time and effectively assesses the severity of proximal pulmonary artery (PA) remodeling and stiffness (Pandian et al., 1990; Borges et al., 1997). In this Research Topic, Grignola et al. demonstrated that both pulmonary arterial capacitance (PAC) and right ventricle-pulmonary artery (RV-PA) coupling were related to PA remodeling through a comprehensive evaluation of IVUS, right heart catheterization (RHC), blood biochemistry, echocardiography, and hemodynamics. Their data raise the possibility that dynamic changes in the pulmonary vascular wall resulting from drugs can be monitored by IVUS in real-time in a clinical trial setting, providing a reliable means for the administration and evaluation of PVD drugs. Due to the wide array of etiologies in the pathogenesis, the combined use of multiple diagnostic tests sheds critical guidance for rapid and accurate diagnosis as well as risk stratification of PAH.

Experimental PH has been primarily modeled with the use of rodents (Sztuka and Jasinska-Stroschein, 2017). Hypoxia-induced pulmonary hypertension (HPH) models develop mild diseases in both rats and mice, while monocrotaline-induced pulmonary hypertension (MCT-PH) in rats is more severe and eventually lethal. In the last 15 years, SU5416/hypoxia-induced severe PH (SuHx) in rats has emerged as a highly translational model with the development of significant PA remodeling with occlusive, plexiform-like lesions. Large animal PH models have also been used for PVD researchers to perform interventions such as cardiac catherization and biopsy. Related to this topic area, Applegate et al. reported that Vorinostat, a histone deacetylase inhibitor, effectively alleviated hypoxia-induced right heart dysfunction and pulmonary arteriosclerosis in a severe neonatal bovine PH model. In their study, early use and moderate dosing of Vorinostat showed a sustained protective effect on the neonatal bovine heart. The pathological characteristics of the hypoxia-induced PH model in newborn calves better mimicked human PAH, emphasizing the translational potential of large pre-clinical animal models (such as calves) for the development of new therapeutic strategies.

Pulmonary artery smooth muscle cells (PASMCs) and pulmonary artery endothelial cells (PAECs) are two widely used cell types for *in vitro* PH studies. Rodriguez et al. provided novel insights into the role of the calcium homeostasis modulators, CALHM1 and 2, in the contractile-to-proliferative phenotypical transition of PASMCs. CALHMs were up-regulated in PAs isolated from MCT-PH rats and PH patients. The up-regulated CALHMs contribute to pulmonary vascular remodeling and PH progression by promoting PASMCs contractile-to-proliferative phenotypical transition through Ca^2+^ influx. This contractile-to-proliferative phenotypical transition could be partially reversed by CALHMs inhibitor in hypoxia-induced PH mice. This medial PASMC transition is characterized by their transformation into a proliferative phenotype and potential for migrating into the intima, resulting in pulmonary artery intima thickening, a hallmark finding in PAH. This work emphasizes the need for more research to elucidate the cellular and molecular mechanisms of this phenotypic switching, although the involvement of calcium signaling pathways, growth factors, inflammation-related pathways, and epigenetic changes is well known. In this Research Topic, another study highlighted aging as a novel potential trigger for PASMCs proliferation. Wang et al. reported a relevance between cellular senescence and proliferation of PASMCs. The increased expression of IL-6 in senescent PASMCs promoted proliferation, which in turn could be attributed to the activation of mTOR/S6K1 pathway under hypoxia. It is well-known that senescent cells contribute to the development of certain diseases through senescence-associated secretory phenotype. However, in PASMCs, cell senescence might be a key therapeutic target for pulmonary artery remodeling. In PAECs, endothelial-to-mesenchymal transition induced by hypoxia-inducible factor 2α is considered to be a significant cause of pulmonary artery media thickening (Tang et al., 2018). In addition, T cells, macrophages, adventitial layer fibroblast cells, extracellular vesicles, and their contents also play roles in the development and progression of PAH. By analyzing the overlapping differential expression genes in two microarray datasets from the lung tissues of PH patients and controls, Yao et al. offer a practical approach for target identification and drug screening. In their study, nine key hub proteins, five key transcriptional factors, and a series of microRNAs related to PAH were reported by gene pathway enrichment analysis and protein-protein interaction analysis, while the affinities of HSP90AA1 protein to nedocromil and SNX-5422 were predicted by protein-drug interaction analysis. Simulating the three-dimensional structure of proteins to yield more targeted drug screening hold promise for research in therapeutics for PAH.

In conclusion, a series of PVD studies ranging from clinical diagnosis of PH to the prediction of targeted drugs have been presented in this special issue. However, gaps in the field demand substantial, additional research progress to extend the understanding of the pathogenesis, and promote the development of diagnostic methods, therapeutic drugs, and clinical management for PVD.

## Author Contributions

JZ, JC, and HT draft the manuscript with input from JW, AD, and SB. All authors contributed to the article and approved the submitted version.

## Funding

This work was supported in parts by National Key Research and Development Program of China (2019YFE0119400) and Natural Science Foundation of China (81970052 and 82170057).

## Conflict of Interest

The authors declare that the research was conducted in the absence of any commercial or financial relationships that could be construed as a potential conflict of interest.

## Publisher's Note

All claims expressed in this article are solely those of the authors and do not necessarily represent those of their affiliated organizations, or those of the publisher, the editors and the reviewers. Any product that may be evaluated in this article, or claim that may be made by its manufacturer, is not guaranteed or endorsed by the publisher.
